# Factors determining the survival or mortality of *Nyctereutes procyonoides* (Canidae) rescued by a wildlife rescue centre: Is urbanisation a threat to wildlife?

**DOI:** 10.3897/BDJ.13.e163708

**Published:** 2025-10-06

**Authors:** Bong Kyun Kim, Soo Hyung Eo

**Affiliations:** 1 Kongju National University, Yesan, Republic of Korea Kongju National University Yesan Republic of Korea; 2 Chungnam Wild Animal Rescue Center, Yesan, Republic of Korea Chungnam Wild Animal Rescue Center Yesan Republic of Korea

**Keywords:** collision with vehicle, raccoon dog, sarcoptic mange, triage, wildlife management, wildlife rehabilitation

## Abstract

Effective wildlife conservation requires an understanding of species' ecological characteristics and identifying survival-threatening factors. Specifically, mitigating anthropogenic threats is imperative for wildlife facing human-wildlife conflicts, such as raccoon dogs (*Nyctereutes procyonoides*). This study analysed data collected over 11 years on raccoon dogs rescued in South Korea, categorising 1,123 rescue cases, based on seven variables. Furthermore, it examined the impact of these variables on incident frequency and subsequent outcomes, including release and mortality. In raccoon dogs, rescue frequency was higher in autumn, in males and in juveniles, with incidents frequently occurring near buildings or involving parasitic infections. Causes of distress and triage were identified as key indicators when evaluating the release potential of rescued raccoon dogs. Additionally, the release potential was found to significantly decrease when they were rescued near roads or buildings or had experienced collisions with vehicles, highlighting urbanisation as a major threat to wildlife. This study provides guidance for the protection and management of raccoon dogs and supports the evaluation and improvement of wildlife rescue, treatment and rehabilitation efforts.

## Introduction

The Wildlife Rescue Centre (WRC) rescues wildlife exposed to various accidents resulting from human activities and provides appropriate treatment, management and rehabilitation to restore their health and facilitate their return to suitable habitats. These activities contribute to the welfare of individual wildlife and maintenance of populations (Tribe and Brown 2000, Molina-López et al. 2017, Kim 2019, Cope et al. 2022). Additionally, WRC analyses the accident history of rescued wildlife to identify threats to species and populations, assesses the levels and trends of damage and collects the necessary data to mitigate the risk of accidents (Kim 2006, Kwok et al. 2021, Cope et al. 2022, Pahuja and Narayan 2023). The collected data can be used to evaluate ecosystem health or establish protection strategies for wildlife species and populations (Sleeman and Clark 2003, Molina-López et al. 2017). Based on the above, WRC is recognised for its contribution to wildlife protection, ecosystem conservation and biodiversity maintenance (Romero et al. 2019, Cope et al. 2022).

Rescued wildlife provides valuable biological and ecological information (Tribe and Brown 2000), including species, sex, age, rescue location, causes of distress, condition, treatment and management history (Molina-López and Darwich 2011, Kwok et al. 2021). The collected information and samples from rescued wildlife can be utilised for research in various fields such as veterinary science, ecology, genetics, molecular biology, toxicology and pathology (Cleaveland et al. 2014). For example, veterinary treatment and management protocols for different fracture types were illustrated through the case of a rescued large-billed crow (*Corvus macrorhynchos* Wagler, 1827) (Kim et al. 2024). DNA analysis of rescued bats yielded the first national record of a particoloured bat (*Vespertilio murinus* Linnaeus, 1758), a species not previously documented in the country (Kim et al. 2025). One study analysed the presence of West Nile Virus infection in rescued birds and assessed the risk of disease emergence and spread (López et al. 2010). Moreover, accurate assessment of injury severity in rescued wildlife has been recognised as a key factor in predicting survival prospects and this understanding has been used to establish practical standards for treatment and management (Molony et al. 2007).

Understanding the causes, frequency and risks of accidents involving rescued wildlife offers important advantages (Kim 2006, Molina-López and Darwich 2011, Kim 2019, Kwok et al. 2021). Representative studies include those that analyse the outcomes of rescued wildlife to examine trends related to accidents (Montesdeoca et al. 2017) or present the criticality of the injuries in predicting survival prospects (Molony et al. 2007). Furthermore, these data can provide information for practical mitigation measures addressing the causes of accidents and provide crucial information for enacting or revising relevant institutions and laws (National Institute of Ecology 2017, Ministry of Government Legislation 2024). However, despite growing recognition of the value of data collected by WRCs, related research and utilisation remain limited (Kwok et al. 2021).

Raccoon dog (*Nyctereutes procyonoides* Gray, 1834) is the only species of the genus *Nyctereutes* within the family Canidae; it primarily inhabits East Asia, being widely distributed from eastern Siberia and northern Indochina to Central Europe (Jo et al. 2018). In Korea, with the extinction of canid species such as the grey wolf (*Canis lupus*) and dhole (*Cuon alpinus*), the raccoon dog remains the only extant member of the family Canidae excluding the red fox (*Vulpes vulpes*), which is currently under restoration programmes (Jo and Baccus 2015). Raccoon dogs, which have a broad range of habitat preferences (Saeki et al. 2007), are amongst the most commonly observed wild mammals in Korea (Choi and Park 2006). However, wildlife habitats have been extensively lost due to industrialisation and urbanisation (Kavzoǧlu 2008). Recently, the increased frequency of the species in urban areas has raised concerns about human-wildlife conflicts, including crop damage, landscape destruction and confrontations with people or dogs during walks (Kim et al. 2024). Urbanisation and habitat reduction have increased conflicts between wildlife, including mammal species and humans, exposing wildlife to various accidents (Kim 2006, Treves et al. 2006). Generally, compared to natural environments such as forests or rivers, urban areas with dense artificial structures pose a higher risk of wildlife accidents, such as collisions with vehicles, habitat fragmentation, isolation in artificial structures and attacks by stray dogs (Kim 2006, Kim 2019, Coronel-Arellano et al. 2021). However, specific causes of distress in raccoon dogs remain largely unknown. Thus far, collisions with vehicles have been regarded as the most significant threat, similar to patterns observed in other mammal species (Choi and Park 2006, Kim 2006, Kim 2019). Therefore, analysing the causes and levels of accidents involving raccoon dogs is urgently needed to develop effective protection and management strategies or policies.

This study examined 11 years (January 2011 to December 2022) of rescue and management efforts of raccoon dogs from the Chungnam Wild Animal Rescue Center in South Korea. The data were classified by year, season, sex, age, environment, causes of distress and triage to examine the occurrence levels and release or mortality status under different conditions. Specifically, we hypothesised that: (1) vehicle collisions, as reported in other mammals, would also represent the most frequent and lethal cause of mortality in raccoon dogs; (2) information collected during rescue, particularly injury level, could serve as reliable indicators for assessing release potential; and (3) accidents associated with urbanisation, including buildings, roads and artificial structures, would directly increase incident frequency and mortality risk. By testing these hypotheses, this study aimed to identify the major threats to raccoon dogs and provide a scientific basis for developing effective management and conservation strategies that promote co-existence with humans.

## Material and methods

### Study area

The study area, Chungnam Province South Korea (approximately 36.52°N, 126.80°E), covered approximately 8,248 km² and was composed of 29.8% agricultural area, 49.2% forest and 21% other environments, including urban and industrial areas (Korean Statistical Information Service 2023). It consists of 15 cities and counties and land-use patterns and population density tend to vary across administrative divisions. As of May 2023, the Province had a population of approximately 2.14 million (Korean Statistical Information Service 2025).

### Frequency of rescues and affecting variables

**Figure 1.**
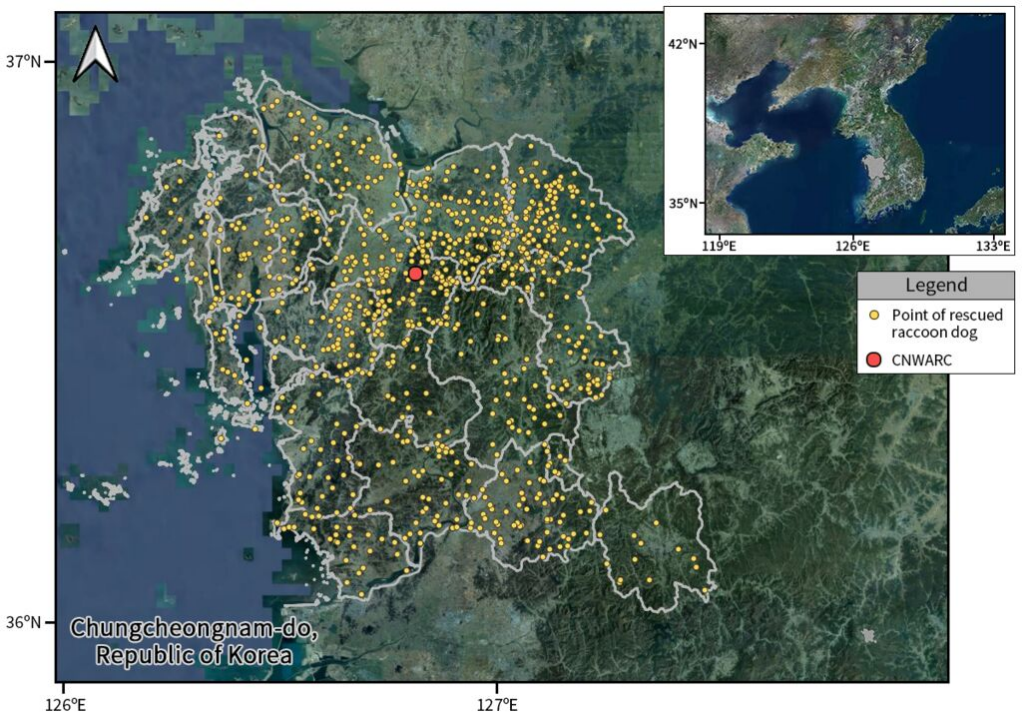
The study area in Chungcheongnam-do, South Korea, where raccoon dog rescues were conducted, along with the actual locations where individuals were rescued (CNWARC: Chungnam Wild Animal Rescue Center).

Between January 2012 and December 2022, the Chungnam Wild Animal Rescue Center in South Korea rescued 1,123 raccoon dogs (Fig. 1). Data obtained from the rescued raccoon dogs were classified, based on six variables: year, season, sex, age, environment and causes of distress (Table 1) to determine how these variables affected accident occurrence. The variables used in the analysis were selected, based on previous studies (Molony et al. 2007, Cope et al. 2022, Pahuja and Narayan 2023). It is worth noting that body weight was considered in the analysis, but ultimately excluded. Although body weight can be an indicator of health, variations in growth levels amongst individuals may complicate analysis and interpretation (Molony et al. 2007).

First, we classified the data by “Year” and “Season”, based on the date the animal was rescued. Annual frequencies were analysed to track temporal changes and trends. Months were classified into four seasons according to the start dates and durations as defined by the Korea Meteorological Administration: spring (March–May), summer (June–August), autumn (September–November) and winter (December–February). Subsequently, the occurrence rates and trends for each season were analysed.

The “sex” of the rescued raccoon dogs was determined by examining the ventral side and identifying the position and shape of the genitalia (Peterson 1988). Classification was marked as unknown in cases where the genitalia were damaged or information was missing, as sex determination was impossible.

“Age” was classified into adult (over one year old), sub-adult (between six months and one year old) and juvenile (under six months old). Individuals whose age could not be determined due to physical damage or missing information were classified as unknown. Age was assessed via radiographic examination to determine the body size and current stage of skeletal development and potential for further growth (Geiger 2015, Rogers et al. 2021). We examined the closure of growth plates in four limb bones: the humerus, ulna, femur and tibia.

“Environment” describes a place where an animal was rescued and is classified into six types: agricultural area, buildings, forest, road, waterbodies and others. "Others" include environments not covered by any of the categories, transitional environments due to construction and mixed environments that are difficult to define, such as outdoor golf courses or parks. The environment was assessed by considering on-site data and satellite images from Google Earth Pro (version 7.3), evaluating the type that occupied the largest area within a 30 m radius and the characteristics of the environment where the accident occurred.

A total of 20 “causes of distress” related to the rescue of raccoon dogs were identified. The five most frequent causes were parasitic infections, collisions with vehicles, orphaning and kidnapping, isolation of artificial structures and entrapment. The remaining 15 less frequent causes were included in the "others" category, making a total of six categories.

### Rescue outcomes and influencing variables

The rescued raccoon dogs were classified into six categories after treatment, rehabilitation and other management: release, dead, dead on arrival (DOA), carcass, euthanasia and in-care. The frequency and proportion of each category were examined. Criteria for each category were as follows: (1) Release, in which individuals were returned to the wild after undergoing treatment and rehabilitation; (2) Dead, in which individuals died during treatment and rehabilitation process; (3) DOA, in which individuals died within 24 hours of being rescued; (4) Carcass, in which individuals were already deceased at the time of admission; (5) Euthanasia, in which individuals were humanely euthanised due to reasons such as disease diagnosis, viral infection or an untreatable condition; (6) In-care, in which individuals were still undergoing treatment or being held long-term for research and educational purposes.

To evaluate the likelihood of release, the categories were further classified into two outcomes: released and not released. The released group comprised individuals who returned to the wild, whereas the non-released group included those who died, were DOA or were euthanised. The remaining outcomes, carcass and in-care, were excluded from this analysis as they did not align to evaluate the factors affecting the release of rescued raccoon dogs.

Subsequently, we added “triage” to the existing six variables to determine how the seven factors affected the likelihood of releasing rescued animals (Table 1). Triage is a concept designed to categorise patients, based on the severity of their injuries, assess treatment potential and assign priority levels (Robertson-Steel 2006). In this study, injuries were classified into four levels based on severity. Level 1 (Normal), indicating no apparent injury; Level 2 (Mild injury), such as minor trauma without weight loss or closed fractures; Level 3 (Severe injury), including open fractures, infected or multiple fractures and clear signs of dehydration and weight loss; and Level 4 (Very severe injury), involving neurological disorders, respiratory impairment, trap-related injuries, blindness, paralysis or severe starvation and exhaustion.

### Statistical analysis

Based on the information collected from rescued raccoons, linear regression and chi-square goodness-of-fit tests were conducted according to the nature of each variable to identify the levels and correlations of accident occurrences across various variables. Additionally, to evaluate the factors influencing the outcomes — release or in-care mortality — of rescued raccoons, logistic regression analyses were performed using categorised variables and the log-odds ratios (ORs) were calculated. ORs, derived through logistic regression analysis, are used to quantitatively assess the extent to which specific conditions affect outcomes (Molony et al. 2007). In this study, the calculated ORs represent the likelihood of release versus in-care mortality for each condition. An odds ratio greater than one indicates that animals influenced by a given variable are more likely to be released compared to those influenced by the reference category, while an odds ratio less than one indicates a higher likelihood of in-care mortality. One variable within each category was set as the intercept and ORs for the remaining variables were compared relative to this reference to quantitatively assess the influence of each factor on the outcome. All statistical analyses conducted in this study were performed using R software (version 4.3.3; R Core Team (2024)).

## Results

### Frequency of rescues and affecting variables

**Figure 2.**
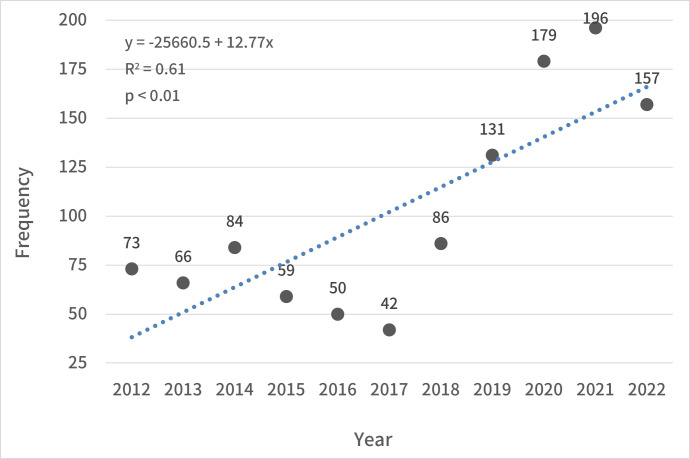
Annual trends in the rescue frequency of raccoon dogs from 2012 to 2022.

An analysis of 1,123 rescued raccoon dogs indicated that they accounted for approximately 25.6% of all rescued mammals during the same period, representing the second-highest proportion after the water deer (*Hydropotes inermis* Swinhoe, 1870). The annual number of rescues increased from 73 in 2012 to 157 in 2022, with an average annual increase of 12.8 individuals (Fig. 2, R² = 0.61, p < 0.01). Seasonal proportions were 24.4, 25.5, 32.3 and 17.8% (274, 286, 363 and 200 cases) in spring, summer, autumn and winter, respectively. The proportion of animals rescued by sex was 48.3% and 37.0% (542 and 416 cases) for males and females, respectively. The proportions by age were 10.5, 16.8 and 25.6% (118, 189 and 288 cases) for adults, subadults and juveniles, respectively. Rescued individuals with unknown sex or age accounted for 14.7% and 47.0% (165 and 528 cases). For environmentals analysis, the highest proportion was observed in buildings (54.1%, 608 cases), followed by agricultural land (19.9%, 224 cases), roads (12.6%, 142 cases), forests (8.2%, 92 cases), waterbodies (2.6%, 29 cases) and others (2.5%, 28 cases). Causes of distress analysis revealed that parasitic infections accounted for the highest proportion, at 60.9% (684 cases). Orphaning and kidnapping were the second-most frequent causes (11.1%, 125 cases), followed by collisions with vehicles (11.0%, 124 cases), isolation in artificial structures (5.3%, 59 cases), entrapment (4.6%, 52 cases) and others (7.0%, 79 cases).

Analysis of the incidence rates of distress causes, based on sex-confirmed individuals, identified parasitic infections as the leading cause in males, accounting for 62.4% (338 cases); orphaning/kidnapping as the second most frequent cause (12.4%, 67 cases), followed by collision with vehicles (10.0%, 54 cases), entrapment (4.8%, 26 cases), isolation from artificial structures (3.7%, 20 cases) and others (6.8%, 37 cases). Amongst the 416 females, parasitic infections were the leading cause, accounting for 62.3% (n = 259). Collision with vehicles was the second most frequent cause (12.3%, 51 cases), followed by orphaning/kidnapping (9.6%, 40 cases), entrapment (4.3%, 18 cases), isolation in artificial structures (3.6%, 15 cases) and other causes (7.9%, 33 cases).

**Figure 3.**
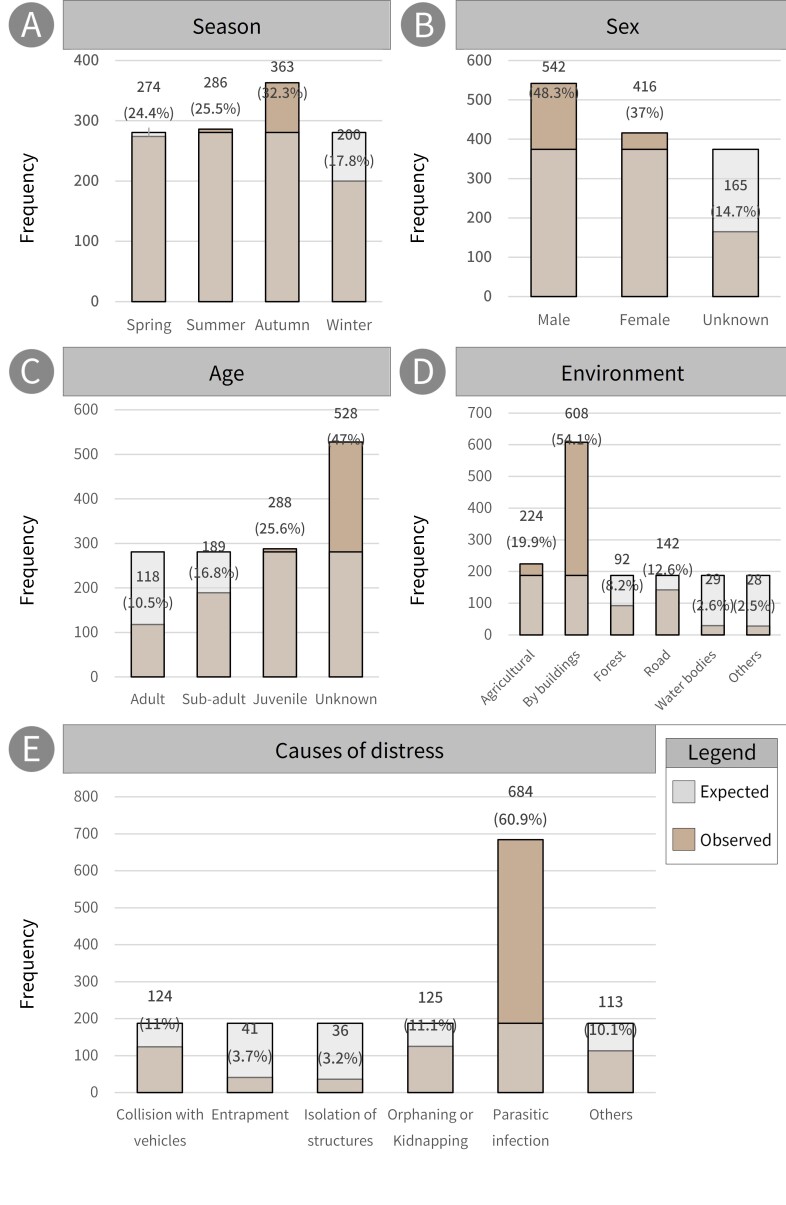
The frequency and proportion of accidents involving raccoon dogs were assessed according to various variables.

Analysis of the incidence rates of the causes of distress, based on age-confirmed individuals, identified parasitic infections as the leading cause of distress amongst adults, accounting for 49.2% (58 cases). Collision with vehicles was the second most frequent cause (17.8%, 21 cases), followed by entrapment (12.7%, 15 cases), isolation in artificial structures (4.2%, 5 cases) and others (16.1%, 19 cases). Amongst sub-adults, parasitic infections were the leading cause, accounting for 75.1% (142 cases). The second most frequent cause was collisions with vehicles (12.2%, 23 cases), followed by entrapment (5.3%, 10 cases), isolation in artificial structures (1.6%, 3 cases) and others (5.8%, 11 cases). Amongst juveniles, parasitic infections, orphaning and kidnapping were identified as the leading causes, accounting for 34.4% (n = 99). The third most frequent cause was collisions with vehicles (12.2%, 35 cases), followed by isolation in artificial structures (7.6%, 22 cases), entrapment (2.1%, 6 cases) and others (9.4%, 27 cases). The frequency of rescues for each variable is shown in Fig. 3.

### Rescue outcomes and correlation of variables

The analysis of outcomes for 1,123 rescued raccoon dogs revealed that release accounted for the highest proportion at 41.2%, followed by dead at 22.3%, DOA at 16.2%, euthanasia at 11%, carcass at 8.5% and in-care at 0.8% as of 1 January 2023.

The analysis of the likelihood of release, based on seasons, showed that, compared to spring, summer had a 2.69 times higher likelihood of release (OR = 2.69, 95% CI = 1.88–3.85, p < 0.001). In contrast, there were no significant differences in the likelihood of release between autumn (OR = 0.74, 95% CI = 0.53–1.04, p > 0.05) and winter (OR = 0.80, 95% CI = 0.54–1.19, p > 0.05) compared to spring.

The evaluation of the likelihood of release, based on sex, showed no significant differences between males and females (OR = 0.85, 95% CI = 0.65–1.11, p > 0.05). However, unknown individuals had a 0.59 times lower likelihood of release compared to males (OR = 0.59, 95% CI = 0.39–0.88, p < 0.05).

In terms of age, the likelihood of release showed that sub-adults had a 0.51 times lower likelihood of release than adults (OR = 0.51, 95% CI = 0.31–0.85, p < 0.01), whereas juveniles had a 2.47 times higher likelihood of release (OR = 2.47, 95% CI = 1.57–3.92, p < 0.001). There was no significant difference in the likelihood of release between unknown individuals and adults (OR = 0.73, 95% CI = 0.48–1.12, p > 0.05).

The analysis of the likelihood of release, based on environment, showed that, compared to agricultural areas, the likelihood of release was 0.63 times lower by buildings (OR = 0.63, 95% CI = 0.46–0.87, p < 0.01) and 0.32 times lower on road (OR = 0.32, 95% CI = 0.19–0.52, p < 0.001). In contrast, there were no significant differences in the likelihood of release from forest (OR = 0.66, 95% CI = 0.39–1.09, p > 0.05), waterbodies (OR = 1.38, 95% CI = 0.61–3.26, p > 0.05) or others (OR = 1.11, 95% CI = 0.49–2.58, p > 0.05) compared with agricultural area.

Evaluating the likelihood of release, based on the causes of distress, demonstrated that, compared to collisions with vehicles, the likelihood of release was 3.38 times higher for entrapment (OR = 3.38, 95% CI = 1.54–7.52, p < 0.01), 52.40 times higher for isolation in artificial structures (OR = 52.40, 95% CI = 14.28–340.90, p < 0.001), 11.35 times higher for orphaning and kidnapping (OR = 11.35, 95% CI = 6.07–22.07, p < 0.001), 2.09 times higher for parasitic infection (OR = 2.09, 95% CI = 1.27–3.56, p < 0.01) and 4.56 times higher for others (OR = 4.56, 95% CI = 2.45–8.76, p < 0.001).

**Figure 4.**
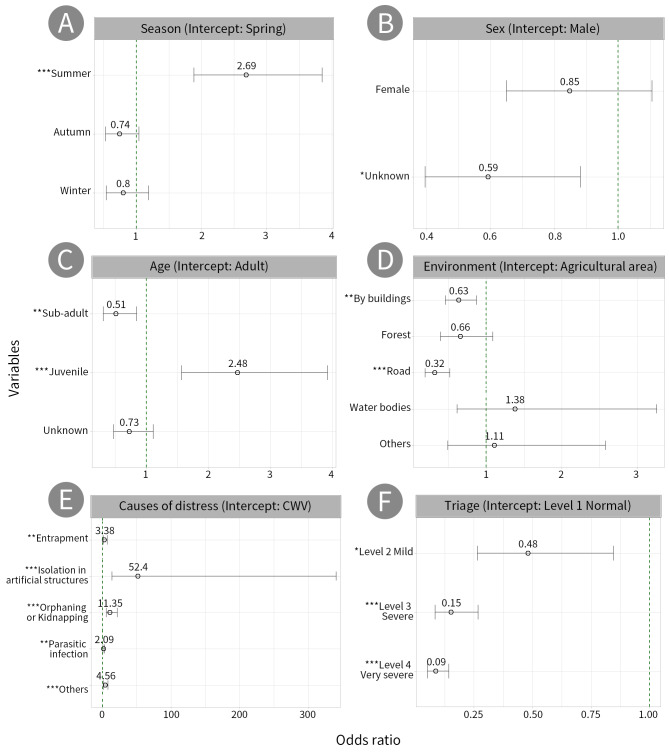
Release rates of rescued raccoon dogs were analysed according to variables using log odds. The green dashed line at 1.0 represents the odds ratio corresponding to the intercept; values above 1 indicate an increased and values below 1 a decreased likelihood of release relative to the intercept. Statistical significance is indicated by asterisks: *p < 0.05, **p < 0.01, ***p < 0.001.

Based on the triage, compared to baseline Level 1, the likelihood of release was 0.48 times lower for Level 2 (OR = 0.48, 95% CI = 0.26–0.85, p < 0.01), 0.15 times lower for Level 3 (OR = 0.15, 95% CI = 0.08–0.27, p < 0.001) and 0.09 times lower for Level 4 (OR = 0.09, 95% CI = 0.05–0.14, p < 0.001), indicating that the more severe the injury, the lower the likelihood of release. The frequency of rescues for each variable is shown in Fig. 4.

## Discussion

A previous study analysing wild mammal rescue outcomes, based on wildlife accident records collected in Chungcheongnam-do over seven years (2011–2017), showed that 26.3% of mammals were released and 72.1% died (Kim 2019). Compared to this, raccoon dogs have a relatively higher likelihood of being released than other wild mammals. Therefore, this species can be considered to have a relatively high recovery potential when active rescue, treatment, rehabilitation and management are appropriately applied. This highlights the importance of implementing active rescue measures to protect and manage raccoon dogs.

Seasonal variation was observed in raccoon dog rescue frequency, with an increase in autumn and a decrease in winter. This pattern may be associated with the species’ life history traits. Juveniles generally undergo natal dispersal and separation from their parents in September and October (Saeki 2001, Drygala and Zoller 2010). During dispersal, individuals entering unfamiliar areas are more vulnerable to accidents and mortality due to difficulties in adapting to new environments (Knowlton et al. 1999). Supporting this, an analysis of age-confirmed rescue cases showed that, excluding individuals of unknown age, juveniles corresponding to the dispersal period were rescued at relatively higher frequencies. In contrast, the decline in rescue frequency during winter may be explained by a seasonal reduction in activity levels. Raccoon dogs tend to reduce activity, remain in shelters and conserve energy during the coldest months and this decreased activity likely lowers their risk of exposure to accidents (Zoller and Drygala 2013).

In certain environments, the proportion of raccoon dog rescues by buildings exceeded half of the total cases. Compared with a previous study that analysed the rescue environments of wild mammals and reported 16.7–23.6% of cases occurring by buildings (Lee 2016, Kim 2022), the proportion observed in raccoon dogs was markedly higher. This result indicates that raccoon dogs utilise urbanised spaces more actively than other species, which is consistent with the recent tendency to classify them as an urban adapter (Kim et al. 2024). However, it also demonstrates that raccoon dogs are highly vulnerable to various anthropogenic threats prevalent in urban areas. Therefore, rather than considering urbanised environments as suitable habitats for raccoon dogs, it is more appropriate to regard them as relatively high-risk environments when compared with their primary habitats, such as agricultural areas and forests.

Amongst the causes of distress threatening this species, parasitic infections caused by scabies mites (*Sarcoptes scabiei* Linnaeus, 1758) were the most frequent. This contrasts with previous studies that analysed the causes and frequencies of distress in wild mammals, in which collisions with vehicles were identified as the most frequent cause (Kim 2006, Kim 2019). Conversely, this finding aligns with studies evaluating the threat factors for raccoon dogs in Japan, where parasitic infections due to scabies mites were identified as significant factors potentially contributing to the population decline (Sugiura et al. 2018, Matsuyama et al. 2024). Therefore, scabies mite infections pose a serious threat to raccoon dogs in South Korea and may significantly contribute to population decline. Notably, parasitic infections were confirmed to have the second lowest likelihood of release amongst the six analysed causes, supporting the notion that treatment is challenging and recovery prospects are low when moderately infected (Kim 2020).

When evaluating seasonality, the likelihood of release was found to be the highest for raccoon dogs rescued in summer. This result is presumed to be related to the birth and rearing periods of the species. In South Korea, raccoon dogs give birth and raise their juveniles from late spring to summer (Jo et al. 2018); during this period, the frequency of rescuing juveniles is high. The high likelihood of releasing juveniles without major injuries is closely related to this result. This is consistent with findings for mammals, particularly canids such as foxes and coyotes, where individuals rescued as orphans showed relatively higher release rates (Hanson et al. 2021, Lukesova et al. 2022, Mullineaux and Pawson 2023). The likelihood of release for individuals rescued in other seasons was relatively low, likely because of the high frequency of rescues resulting from parasitic infections.

No difference was observed in the likelihood of release, based on the sex of the species. However, age showed a difference, with the likelihood of release being the highest for juveniles. The primary causes of distress amongst juveniles were orphaning and kidnapping. Those rescued due to this cause often do not have physical injuries or disabilities. Therefore, it is inferred that the higher likelihood of release compared with other age groups is due to this reason. These results suggest that, if WRCs actively engage in juvenile rescue, positive outcomes can be achieved in terms of protection and management. However, caution is necessary, as hasty rescue without confirming the presence or care of parents may interfere with the normal upbringing process of the population (Kim 2019).

The likelihood of release was relatively low for raccoon dogs found near roads and buildings. This suggests that accidents near roads and buildings pose a significant threat to this species. The results of this study indicate that urban areas are frequently utilised as habitats for raccoon dogs and that roads and artificial structures negatively affect their survival. Based on this, if the environment constituting the habitat of the species has a high proportion of roads and artificial structures, that is, highly urbanised areas, the habitat can be considered a high-risk environment due to the high likelihood of accidents. Additionally, when a WRC releases this species back into the wild, it is necessary to fully consider the area and accessibility of roads and artificial surfaces when evaluating and selecting new habitats.

Amongst the causes of distress, the likelihood of release was the lowest for species involved in collisions with vehicles. The severity and high frequency of these accidents support previous findings that this is the most direct and significant threat to wild mammals (Kim 2006, Park et al. 2012, Kim 2019, Paredes-Casas et al. 2024). Additionally, when combined with the results of this study, which analysed the likelihood of release based on environmental types, it re-affirmed that roads, which are environments where vehicle collisions frequently occur, pose a high risk to raccoon dogs.

Analysis of the severity of injury at the time of rescue demonstrated that raccoon dogs with more severe injuries had a higher mortality rate. This indicates that current diagnostic methods and criteria for assessing injury levels are appropriate. Additionally, this finding is consistent with previous research that identified injury level as a major variable in predicting the outcome of rescued wildlife (Molony et al. 2007). Therefore, the injury level of individuals must be actively considered in their treatment and management. Specifically, actively using the injury level to evaluate the necessity of euthanasia is crucial to prevent the welfare deterioration of individuals through unnecessary treatment and management of those who are difficult to recover. The types and levels of damage inflicted on individuals vary significantly depending on the causes of distress that threaten the wildlife (Molony et al. 2007, Kim 2020, Cope et al. 2022). Therefore, accurately assessing injury levels in rescued animals using appropriate diagnostics is crucial. Furthermore, it is crucial to consider injury levels when establishing treatment protocols or determining the likelihood of release. The results of this study can be actively utilised to accumulate quantitative data on the protection and management of raccoon dogs, as well as to evaluate and improve the appropriateness of wildlife rescue, treatment, rehabilitation and management systems. Moreover, the study provides a basis of management and conservation measures for the co-existence of raccoon dogs and humans, as well as potential for future studies on the damage to wildlife and habitats due to urbanisation, contributing to biodiversity conservation.

## Conclusions

Based on the analysis of habitat status and the factors influencing the release and mortality of raccoon dogs rescued by the Chungnam Wildlife Rescue Center, parasitic infections caused by scabies mites were identified as the most frequent cause of distress. This finding contrasts not only with previous studies on wild mammals, which reported vehicle collisions as the most common cause of distress, but also with our initial hypothesis that vehicle collisions would represent the greatest threat to raccoon dogs. However, collisions with vehicles were still identified as the second most frequent cause, re-affirming their significance as a serious threat to the species.

Amongst the variables analysed to predict the likelihood of release, the severity of injury and the cause of distress emerged as key determinants. Utilising these variables may enable the prioritisation of treatment for rescued individuals, efficient allocation of medical and management resources and the establishment of appropriate euthanasia criteria. Additionally, raccoon dogs rescued near roads or buildings or those involved in vehicle collisions, were found to have a markedly higher likelihood of mortality.

These results suggest that, despite the species' ecological adaptability and capacity to survive in urban environments, habitat changes driven by urbanisation pose a substantial threat to raccoon dogs. Therefore, when considering the release of rescued raccoon dogs back into the wild, it is crucial for wildlife rescue and management centres to carefully evaluate the density and accessibility of roads and artificial structures in the candidate habitats. Such considerations will help ensure the long-term survival and conservation of this species in increasingly human-dominated landscapes.

## Figures and Tables

**Table 1.** Description of the variables used in the analysis for raccoon dogs (*Nyctereutes procyonoides*) admitted to Chungnam Wild Animal Rescue Center.

Variable	Variable type	Description
Year	Categorical	From 2012 to 2022
Season	Categorical	Spring (Mar–May), Summer (Jun–Aug), Autumn (Sep–Nov), Winter (Dec–Feb)
Sex	Categorical	Male, Female, Unknown
Age	Categorical	Adult, Sub adult, Juvenile, Unknown
Environment	Categorical	Agricultural area, By buildings, Forest, Road, Waterbodies, Others
Causes of distress	Categorical	Six distinct causes of distress and an "Others” group (comprising 15 additional causes)
Triage (Level of injuries)	Categorical	Level 1 (Normal), Level 2 (Mild injury), Level 3 (Severe injury), Level 4 (Very severe injury)
Outcome	Categorical	Dependent variable: released or non-released
